# Anthelmintic Resistance in Livestock Farming: Challenges and Perceptions of Farmers and Veterinarians

**DOI:** 10.3390/pathogens14070649

**Published:** 2025-06-30

**Authors:** Naida Kapo, Adis Softić, Teufik Goletić, Šejla Goletić, Aleksandar Cvetkovikj, Jasmin Omeragić

**Affiliations:** 1 University of Sarajevo-Veterinary Faculty, Zmaja od Bosne 90, 71000 Sarajevo, Bosnia and Herzegovina; adis.softic@vfs.unsa.ba (A.S.); teufik.goletic@vfs.unsa.ba (T.G.); sejla.goletic@vfs.unsa.ba (Š.G.); jasmin.omeragic@vfs.unsa.ba (J.O.); 2 Faculty of Veterinary Medicine Skopje, National Veterinary and Food Institute, Ss. Cyril and Methodius University in Skopje, 1000 Skopje, North Macedonia; acvetkovic@fvm.ukim.edu.mk

**Keywords:** self-reported data, questionnaire, anthelmintic resistance, farmers, veterinarians, parasite control, Bosnia and Herzegovina

## Abstract

Anthelmintic resistance in livestock is a growing concern worldwide, with significant implications for animal health and agricultural productivity. This study explores the perceptions of veterinarians and farmers in Bosnia and Herzegovina regarding the factors contributing to anthelmintic resistance in *Haemonchus contortus* nematodes. Data were collected through structured questionnaires completed by 106 veterinarians and 188 farmers in 2022 and 2023. The analysis focused on self-reported therapeutic practices, farm management and environmental variables. Logistic regression, including Firth’s penalized approach, was used to assess associations between these perceived factors and the reported occurrence of resistance. Notably, combination anthelmintic treatments were perceived as a significant risk factor (OR > 49.3), while higher altitude was seen as potentially protective (OR = 0.10). Routine prophylactic deworming was associated with an increased likelihood of perceived resistance (OR = 173.7), whereas staying informed about newly registered products was perceived as protective (OR = 0.34). Although the findings are based on the self-reported perceptions and practices of veterinarians and farmers, they align with globally recognized trends and offer the first structured insights into factors perceived to contribute to anthelmintic resistance in Bosnia and Herzegovina. This study underscores the importance of awareness and responsible anthelmintic use and the need for improved diagnostics and ongoing education to combat anthelmintic resistance.

## 1. Introduction

Gastrointestinal nematode (GIN) infections are among the most common parasitic diseases in ruminants, causing substantial economic losses and mortality in sheep, goat and cattle farming worldwide [1]. Among GIN species, *Haemonchus* spp. are frequently detected and have a particularly significant impact on production, contributing to major losses, reduced growth and increased susceptibility to other diseases [2]. For more than 50 years, parasite control strategies have relied primarily on the frequent use of broad-spectrum anthelmintics, which were initially highly effective [3]. However, the efficacy of these treatments is now jeopardized by the emergence of nematode populations resistant to one or more available anthelmintic classes. The increasing prevalence of anthelmintic resistance (AR) in GIN globally, along with the limited number of effective drugs, continues to escalate production costs and poses long-term challenges for livestock sustainability. The rate at which AR develops depends on multiple factors, including parasite biology [4,5] and farm-level practices such as treatment frequency, incorrect dosing (under-dosing, mass medication) and how anthelmintics are administered. The combination of these factors may accelerate the establishment of AR in specific regions depending on local deworming practices [6].

In Bosnia and Herzegovina, the issue of benzimidazole (BZ) resistance in *Haemonchus contortus* had not been investigated until recently. A molecular study using real-time qPCR detected the F200Y single-nucleotide polymorphism (SNP) in *H. contortus* isolated from sheep, goats and cattle, marking the first report of this mutation in the country [7]. The results showed that 86.8% of isolates were homozygous resistant, 8.4% heterozygous resistant and only 4.8% homozygous susceptible at codon 200 of the β-tubulin gene. Resistance was widespread, with homozygous resistant genotypes found in 100% of goats, 77.4% of sheep and 94.7% of cattle. These findings suggest that the F200Y mutation is well established in *H. contortus* in ruminant populations across Bosnia and Herzegovina [8]. Given the common use of BZs for deworming and the practice of grazing sheep, goats and cattle together on shared pastures, these results raise significant concerns about cross-species transmission and the further spread of resistance [8]. Transhumance and animal movement were also identified as contributing factors to the dissemination of resistant parasites, reflecting patterns observed in other parts of Europe [9,10,11].

Therefore, understanding the deworming practices of farmers and veterinarians is essential to evaluate their role in the development and spread of AR. In Bosnia and Herzegovina, there is currently no standardized treatment protocol for haemonchosis, despite its well-documented impact on livestock production. Furthermore, the ability of farmers to purchase anthelmintics without veterinary consultation highlights the risk of inappropriate treatments and increases the potential for resistance development [8].

Survey-based studies of GIN control practices among livestock holders have proven useful for understanding the behaviors and decision-making processes of both farmers and veterinarians. However, in Bosnia and Herzegovina, little is known about how parasite control and AR are approached by these stakeholders.

This study was conducted to assess the deworming practices of livestock farmers and veterinarians in relation to parasite control and AR in Bosnia and Herzegovina, with the aim of informing the development and implementation of sustainable parasite control strategies. The findings also emphasize the urgent need to strengthen access to diagnostics and provide continuous education on the proper use of anthelmintics.

## 2. Materials and Methods

### 2.1. Data Collection and Variables

Bosnia and Herzegovina covers an area of approximately 51,000 square kilometers and has a population of around 3.3 million. The country features a variety of climatic zones, ranging from a temperate continental climate in the inland regions to a Mediterranean climate in the south, with annual precipitation between 800 and 1200 mm. Livestock production is largely based on family-owned farms operating under extensive and semi-extensive husbandry systems, which often include seasonal transhumance and nomadic grazing practices. According to available data, the country is home to approximately 82,000 cattle, 1,000,000 sheep and 60,000 goats.

During 2022 and 2023, extensive testing for AR in *H. contortus* nematodes was conducted across Bosnia and Herzegovina [7]. As part of this study, parasite samples were collected from farms located in five geographic regions: western, southwestern, central, northeastern and eastern Bosnia and Herzegovina. In parallel with laboratory testing, structured questionnaires were administered to farmers and veterinarians responsible for health monitoring on the sampled farms.

It was hypothesized that most farmers were not familiar with the concept of AR and that their level of knowledge, attitudes and practices related to AR would be insufficient.

In collaboration with local veterinary clinics and field veterinarians, farmers and veterinarians involved in sample collection were provided with background information about the study and its objectives. To reduce non-response and bias in answers to sensitive questions, participants were assured that their responses would remain anonymous. In total, 188 farmers and 106 veterinarians agreed to participate in the structured interviews.

Data were collected using structured questionnaires administered to both veterinarians and farmers capturing their perceptions and self-reported practices related to anthelmintic usage (e.g., albendazole, combinations, frequency), management practices (e.g., quarantine of new animals, introduction of new livestock) and environmental factors (e.g., altitude of the farm) (see Appendix A Table A1 and Table A2). To confirm the validity of the questionnaire, it was piloted in 2021 among a small group of 10 farmers and 5 veterinarians. Questions were revised for clarity based on feedback. Face validity was established through expert review by three senior parasitologists. To minimize non-response and bias, anonymity was guaranteed. Variables were coded according to a predefined codebook (see Appendix A Table A3 and Table A4), and resistance to treatment (present/absent) was treated as a binary outcome.

### 2.2. Descriptive Analysis

Descriptive statistics were calculated to summarize sample characteristics and explore patterns in the data. Categorical variables were described using frequencies and percentages, and bivariate tables were generated to observe the distribution of resistance across categories.

### 2.3. Logistic Regression: Model Framework

Logistic regression was used to estimate the probability of resistance based on explanatory variables. Logistic regression estimates the probability of a binary outcome *Y* (resistance: 1 = present, 0 = absent) as a function of one or more explanatory variables: X1, X2, …, XkX_1, X_2, …, X_kX1, X2, …, Xk.

The model takes the following form:

log(1 − P(Y = 1)P(Y = 1)) = β0 + β1X1 + β2X2 + … + βkXk

where

P(Y = 1) is the probability of resistance being present;

β0 is the intercept;

β1, β2, …, βk are the coefficients for predictor variables.

Odds ratios (ORs) were derived as OR = eβ with corresponding 95% confidence intervals (CIs) reported for interpretability.

### 2.4. Firth’s Penalized Logistic Regression: Justification and Use

In the presence of rare events or complete/quasi-complete separation—a common issue in field epidemiological data—traditional maximum likelihood estimation (MLE) can yield biased or infinite estimates. To overcome this, Firth’s penalized likelihood logistic regression was employed using the firthlogit command in STATA/SE 15 (StataCorp, College Station, TX, USA). This method adjusts the score function by introducing a penalty term based on the Jeffreys invariant prior, effectively reducing small-sample bias.

### 2.5. Model Development Strategy

The modeling proceeded in the following stages:

Univariate analysis: each predictor was first tested independently using both standard and Firth’s logistic regression to screen for associations (*p* < 0.25 used for inclusion into multivariable models).

Multivariable modeling: three conceptual models were constructed:

Model 1—therapeutic variables only (e.g., specific drugs, combinations);

Model 2—management and environmental variables (e.g., altitude, quarantine);

Model 3—integrative model combining clinically and statistically relevant variables from Models 1 and 2.

Model selection: a full model with all predictors was subject to backward elimination (using Firth’s logistic regression), systematically removing variables with the highest non-significant *p*-values (*p* > 0.5) to derive a parsimonious and interpretable final model.

### 2.6. Statistical Tools and Thresholds

All statistical analyses were conducted using Stata/SE 15, employing the logit and firthlogit commands. The significance level was set at α = 0.05, though trends (*p* < 0.10) were also discussed where relevant. Model diagnostics, including likelihood convergence and confidence interval width, were assessed to ensure stability.

Prior to conducting regression analyses, several data quality and diagnostic procedures were carried out to ensure the robustness of the models and the reliability of the estimates.

### 2.7. Detection of Outliers

Summary statistics including minimum, maximum, interquartile range and percentiles were inspected using the summarize, detail command in Stata. Additionally, box-and-whisker plots (graph box) were constructed to visually identify extreme values across variables. Outliers were not removed unless they were found to be data entry errors or implausible values based on biological context.

### 2.8. Assessment of Multicollinearity

Given the inclusion of multiple explanatory variables in the multivariable models, the potential for multicollinearity was evaluated using the Variance Inflation Factor (VIF). A VIF threshold of >10 was considered indicative of problematic multicollinearity. In our dataset, all VIF values were below this threshold, suggesting no serious collinearity among predictors.

### 2.9. Distributional Assessment of Continuous Variables

Although logistic regression does not require normality of predictors, we explored the distribution of continuous variables to aid interpretation and understand data behavior. Normality was assessed visually through histograms and statistically using the Shapiro–Wilk test (swilk command in Stata). This allowed us to better contextualize the spread and potential skewness of predictor variables. These diagnostic steps supported the decision to proceed with logistic regression modeling, and no substantial data transformations were deemed necessary.

## 3. Results

### 3.1. Basic Information About Respondents

Basic demographic and professional information about the respondents is presented in Table 1 and Table 2. A total of 188 farmers and 106 veterinarians participated in the survey.

The majority of farmers who responded were over 51 years of age, primarily raising sheep (36.18%) and originating from the northeastern region of Bosnia and Herzegovina (23.94%), which predominantly includes lowland areas (58.51%). A significant proportion of farmers (55.32%) reported practicing transhumance, moving livestock between different grazing locations.

Veterinarians who participated in the survey were mostly between 36 and 50 years of age (50.94%) and represented various parts of Bosnia and Herzegovina. Similarly to the farmers, the highest proportion of veterinarians (27.36%) were also based in the northeastern region. Most were employed at public veterinary stations (66.98%) and had between 16 and 20 years of professional experience (40.57%). The majority of veterinarians (92.45%) reported being familiar with the term anthelmintic resistance.

### 3.2. Interpretation of Models

#### 3.2.1. Model 1: Therapeutic Practices

In the first model, which focused on therapeutic interventions, the use of combination anthelmintic treatments (combi) emerged as a strong potential risk factor for resistance, with an OR of 49.3. Although the association was not statistically significant (*p* = 0.310), the direction and magnitude of the effect suggest a concerning trend (Table 3). This may reflect the selective pressure exerted by multiple drug classes when used simultaneously, possibly accelerating resistance development.

The use of albendazole alone (alben) and albendazole in combination with albendazole ganadexil 10% (albgan) were associated with lower odds of resistance (OR < 1), suggesting possible protective effects, though these findings were not statistically conclusive. Other variables, such as frequency of treatment and previous resistance suspicion (nuth, freq), did not show meaningful associations in this model (Table 3).

#### 3.2.2. Model 2: Management and Environmental Factors

Model 2 explored biosecurity and farm-level practices. Notably, altitude showed a consistent inverse relationship with resistance, particularly for farms situated at higher altitudes (altitude = 3, OR = 0.10; *p* = 0.067) (Table 4). This suggests that ecological and environmental factors may influence parasite pressure or drug efficacy, potentially due to lower stocking densities or reduced transmission dynamics in mountainous regions.

Other factors such as quarantine measures (qarant), the introduction of new animals (newdang) and the person responsible for treatment decisions (which) were not significantly associated with resistance but were included due to their conceptual importance (Table 4).

#### 3.2.3. Model 3: Final Therapeutic Model

In the refined therapeutic model (Model 3), the combination treatment variable (combi) remained a strong and statistically significant predictor (OR = 102.2, *p* = 0.008) (Table 5 and Table 6). This reinforces concerns about the overuse or inappropriate use of combination therapies without proper rotation or diagnostics.

Altitude again demonstrated a protective trend (altitude = 3, OR = 0.10; *p* = 0.072), aligning with findings from Model 2. The variable albendazole (alben) retained its inverse association (OR = 0.092), though it did not reach statistical significance (*p* = 0.117) (Table 5 and Table 6).

### 3.3. Univariate Analysis

Several variables were found to have potential associations with the outcome, notably the following:

sex: male veterinarians were borderline significantly less likely to report reduced effectiveness (OR < 1, *p* = 0.055).

ahreg: tracking updates to registered anthelmintics was associated with lower perceived resistance (*p* = 0.053).

ahrum: veterinarians routinely administering anthelmintics had significantly higher odds of perceiving resistance (OR = 3.29, *p* = 0.032).

dose: approximate dosing practices trended toward increased odds of resistance (*p* = 0.127).

exp and freqyear: these variables showed positive but non-significant trends (*p* = 0.110 and *p* = 0.128).

### 3.4. Multivariable Analysis

The full model included the variables sex, exp, ahreg, ahrum, dose and freqyear. The VIFs were low (mean VIF = 1.10), indicating no collinearity concerns. In the final Firth’s logistic regression model, the following occurred:

ahrum (routine administration by veterinarians) remained significantly associated with higher odds of reported reduced efficacy (OR = 173.7; *p* = 0.008);

ahreg was inversely associated with the outcome (OR = 0.34; *p* = 0.053);

exp (years of experience) was positively associated, though not statistically significant (OR = 1.92; *p* = 0.072).

A simplified model was developed using backward elimination, retaining ahrum, ahreg and exp, yielding a parsimonious model (*p* = 0.0254) (Table 7).

## 4. Discussion

Gaining insight into the factors influencing the decisions of veterinarians and farmers is crucial for developing sustainable parasite control strategies. In this study, the knowledge, attitudes and practices of veterinarians and farmers in Bosnia and Herzegovina regarding parasitic diseases and mitigating AR were assessed through descriptive statistics and qualitative analysis of data collected via questionnaires. While the data indicate a high level of awareness of the AR issue, particularly among veterinarians, they also highlight the need for further education and improvement in practical methods for anthelmintic application, especially among farmers.

Although this study relies on self-reported data, the results are supported by statistically rigorous analyses, providing scientifically grounded insights despite the inherent limitations of subjective reporting. Importantly, only the perceptions and reported practices of veterinarians and farmers were assessed, which limits the ability to directly link practices to verified resistance patterns. Nevertheless, these findings offer a valuable understanding of stakeholder behavior and serve as a foundation for targeted interventions. To the best of our knowledge, this is the first study of its kind conducted in Bosnia and Herzegovina, contributing region-specific data to the broader understanding of AR management.

The sample of veterinarians included in this study demonstrated a high level of awareness regarding AR, with 92.45% of respondents reporting familiarity with the issue. This finding aligns with the quantitative analysis, where routine anthelmintic administration and awareness of newly registered products (ahrum and ahreg) were shown to significantly influence perceptions of treatment efficacy and the emergence of resistance. The consistent association between routine administration of anthelmintics (ahrum) and perceived loss of efficacy suggests that clinical exposure to high treatment frequencies may sensitize veterinarians to detecting resistance trends. Alternatively, it may reflect overuse patterns or selection pressure in practices where veterinarians frequently treat without diagnostics. The inverse association with ahreg highlights the potential value of staying informed on newly registered products and adapting practices accordingly. This suggests that veterinarians who engage with current regulatory updates may adopt more strategic or diversified treatment protocols, potentially mitigating AR. Nevertheless, it is noteworthy that 7.55% of respondents (n = 8) reported unfamiliarity with the term anthelmintic resistance. Although this represents a small proportion of the sample, it raises concerns regarding potential gaps in continuing education or access to up-to-date information. These individuals may represent practitioners who have yet to encounter AR in practice or, conversely, those who have limited engagement in professional development activities. Demographic data provide context for understanding the factors that shape veterinarians’ practices in Bosnia and Herzegovina. The majority of respondents (73.58%) were male, with the largest age group being 36–50 years (50.94%), indicating a professionally mature population. A substantial proportion of the participants (40.57%) had 16 or more years of professional experience, with 17.92% having 21–30 years and 17.92% having over 30 years of experience. While the variable “experience” was not statistically significant, the observed trend (OR > 1) suggests that more experienced practitioners may be better able to recognize resistance patterns, aligning with findings from previous studies. Interestingly, veterinarians employed in the public sector outnumbered those in the private sector by a ratio of 2:1 (66.98% vs. 33.02%), which may influence access to continuing education, diagnostic resources and decision-making processes regarding anthelmintic use. The respondents were also geographically distributed across the country, with the highest proportions from northeastern Bosnia and Herzegovina (27.36%) and the central region (21.7%). These areas are known for more intensive livestock production, which could influence the frequency of anthelmintic use and the potential emergence of resistance. Although the variable “experience” was not statistically significant, its trend aligns with the literature suggesting that experienced practitioners may be more adept at recognizing resistance patterns. However, further research is needed to determine whether this reflects true detection abilities or varying interpretation thresholds based on experience.

Variables such as sex, dose and frequency were not retained in the final model but showed borderline significance or conceptual relevance. Notably, approximate dosing (dose) could contribute to subtherapeutic exposures, which are a recognized factor contributing to resistance. The use of Firth’s regression proved essential for valid inference, particularly in the presence of small sample sizes and quasi-complete separation. This statistical approach ensured more reliable results, even when some categories of data were under-represented. Farmers participating in this study were predominantly middle-aged, with the majority over 50 years old. Despite extensive experience, neither the farmers’ age nor the type of livestock they raised had a significant influence on their understanding of AR. This finding contrasts with other studies, such as those by Jafari et al. [12], which suggest that farmers managing larger herds tend to have better knowledge and awareness of infectious diseases. However, it appears that farmers are generally well informed about diseases presenting visible clinical signs (e.g., lameness or skin lesions) but less so about subclinical conditions like haemonchosis, which is exacerbated by AR.

While herd size (nums) was not statistically significant, the trend (OR > 1), combined with evidence from the literature, suggests that larger herds, due to higher stocking densities and increased parasite pressure, may lead to a higher awareness of the need for parasite control. Larger farms often require more extensive measures to mitigate production losses and manage chemical control reliance. Willock et al. [13] also highlighted farm size as a key determinant in farmers’ decision-making processes regarding parasite control. A consistent association was observed between the use of combination anthelmintic treatments (combi) and increased odds of resistance, suggesting that such treatments may accelerate the selection pressure on parasite populations. The final model showed that this variable remained statistically significant (OR = 98.8; *p* = 0.008). This is consistent with findings from Vadlejch et al. [14] and Mickiewicz et al. [15], who warned against the uncritical use of combination therapies and over-reliance on BZ-based treatments, both of which have been strongly linked to resistance development. Improper use of combination treatments, without diagnostic support, may amplify the survival of resistant genotypes, highlighting the necessity for evidence-based deworming strategies guided by fecal egg counts and farm-specific risk assessments. Although combination therapies were associated with higher odds of resistance, the wide confidence intervals reflect uncertainty due to sample size and should be interpreted with caution. The use of albendazole (alben) demonstrated an inverse association with resistance, although not statistically significant. This trend may reflect more targeted use of single-drug regimens, although emerging genetic mutations conferring BZ resistance serve as a reminder that resistance can spread even with the use of single agents [16]. This underscores the importance of rotating drug classes and avoiding exclusive reliance on any one anthelmintic group. The adoption of strategies such as Targeted Selective Treatment (TST) and Treating All Animals (TT) at specific times can reduce treatment frequency and slow the development of resistance. These approaches, combined with multi-drug formulations, offer a sustainable strategy to manage AR. However, the success of such strategies depends on farmers’ ability to collect samples and the availability of affordable diagnostic tools for parasitological diagnosis before and after treatment [17]. According to self-reported data from farmers, altitude emerged as a potential protective factor against resistance. Farms located at higher elevations (altitude level 3) had markedly lower odds of resistance (OR ≈ 0.10; *p* ~ 0.07), likely due to differences in climate, grazing pressure and stocking density. Similar findings by Solomon et al. [18] suggest that extensive systems with less intensive animal contact are associated with lower parasite burdens. However, mountain farming systems present unique challenges. Routine whole-flock treatments, often administered without coprological analysis, and minimal rotation of anthelmintic classes contribute to resistance. Furthermore, communal grazing on unregulated alpine pastures impedes coordinated parasite control efforts, increasing the risk of resistance spread. Thus, altitude has a dual effect. While reduced infection pressure may limit parasite transmission, infrastructural constraints in mountainous regions can hinder effective parasite management. These findings underscore the importance of region-specific strategies for managing GIN infections and preserving the efficacy of anthelmintics. Variables such as the quarantine of new animals (qarant) and the introduction of new stock (newdang) did not show statistically significant associations but were included due to their biological plausibility. The introduction of resistant worms through the purchase of live animals has been documented in several European countries [19,20]. Ensuring high biosecurity and performing thorough parasitological checks before introducing new animals to pastures are essential to reduce the spread of resistance. Implementing effective quarantine protocols is crucial, as is reducing the movement of animals between farms [21]. Artificial insemination is encouraged where possible to reduce the need for animal movement. The demographic profile of farmers in this study was strongly skewed toward older age groups, with 84.04% of respondents over the age of 50. This may present challenges for adopting new parasite control practices, particularly sustainable anthelmintic use strategies. The age structure may partially explain the continued use of traditional livestock management practices, including conventional anthelmintic regimens. Farm distribution was geographically diverse, with the highest proportion of farms in northeastern Bosnia and Herzegovina (23.94%), followed by the central (18.09%) and southwestern regions (17.02%). These regions are known for significant livestock production, making the findings representative of the broader national context. Grazing conditions, climatic factors and ecological pressures, such as farming in lowland (58.51%) and hilly areas (35.64%), influence parasite dynamics and the effectiveness of control measures. In terms of livestock type, most farmers raised sheep (36.17%), followed by cattle (30.32%) and goats (26.60%), reflecting the mixed livestock production system in Bosnia and Herzegovina. The diverse livestock combinations, including sheep and goats (5.85%) or sheep and cattle (1.06%), are relevant in the context of cross-species parasite transmission, which can complicate infection control and elevate the risk of resistance development. Seasonal grazing (55.32%) and indoor housing (36.7%) were the most common management practices. While seasonal grazing can reduce parasite exposure, indoor housing, if not properly managed, can facilitate the accumulation of parasites in the environment. Herd sizes were generally small to medium, with 26.6% of farms maintaining 1–10 animals and an equal proportion with 10–20 animals. Only 15.43% of farms had more than 40 animals, indicating limited capacity for monitoring animal health and ensuring consistent anthelmintic treatment.

## 5. Conclusions

This study provides the first comprehensive assessment of farmers’ and veterinarians’ knowledge, attitudes and practices regarding AR in Bosnia and Herzegovina. It is important to emphasize that the conclusions are based on survey data reflecting the perceptions of farmers and veterinarians, which may not always fully represent the actual epidemiological situation. Therefore, all data expressed as ORs represent subjective assessments and should be interpreted within this context. Nevertheless, survey-based research offers valuable insights into key factors influencing AR development, such as the increased risk associated with combination treatments and the potential protective effect observed in farms located at higher altitudes. Routine anthelmintic use correlated with higher resistance levels, while veterinarians maintaining up-to-date knowledge on new drugs were associated with a lower risk of contributing to AR emergence. These findings stress the urgent need to raise farmer awareness, improve access to affordable diagnostics and promote evidence-based, targeted deworming education. Future efforts should focus on strengthening veterinary diagnostic support, continuous education and access to updated regulatory information. Overall, integrating data from both veterinarians and farmers is essential to effectively address AR and improve parasite control strategies.

## Figures and Tables

**Table 1:** General characteristics of farmers (n = 188) in study on deworming practices and anthelmintic resistance in Bosnia and Herzegovina, 2024.

General Characteristics	Number (%)
Age (years)	
18–35	6 (3.19)
36–50	24 (12.77)
51–65	79 (42.02)
Over 65	79 (42.02)
Farm Location (Geographical Area)	
Western Bosnia and Herzegovina	23 (12.23)
Southwestern Bosnia and Herzegovina	32 (17.02)
Central Bosnia and Herzegovina	34 (18.09)
Northeastern Bosnia and Herzegovina	45 (23.94)
Eastern Bosnia and Herzegovina	25 (13.27)
Herzegovina	29 (15.43)
Farm Location (Elevation Type)	
Lowland	110 (58.51)
Hill	67 (35.64)
Mountain	11 (5.85)
Type of livestock	
Sheep	68 (36.17)
Goat	50 (26.60)
Cattle	57 (30.32)
Sheep and goat	11 (5.85)
Sheep and cattle	2 (1.06)
Grazing practices	
Permanent grazing	15 (7.98)
Seasonal grazing	104 (55.32)
Enclosed facility with or without an outdoor area	69 (36.7)
Herd size	
1–10	50 (26.6)
10–20	50 (26.6)
20–30	44 (23.4)
30–40	15 (7.98)
>40	29 (15.43)

**Table 2:** General characteristics of veterinarians (n = 106) in study on deworming practices and anthelmintic resistance in Bosnia and Herzegovina, 2024.

General Characteristics	Number (%)
Gender	
Male	78 (73.58)
Female	28 (26.42)
Prefer not to specify	0 (0)
Age (years)	
18–35	16 (15.09)
36–50	54 (50.94)
51–65	28 (26.42)
Over 65	8 (7.55)
Years of experience	
0–4	6 (5.66)
5–15	19 (17.92)
16–20	43 (40.57)
21–30	19 (17.92)
Over 30	19 (17.92)
Type of veterinary organization	
Private	35 (33.02)
Public	71 (66.98)
Canton/county of veterinary organization	
Western Bosnia and Herzegovina	21 (19.81)
Southwestern Bosnia and Herzegovina	15 (14.15)
Central Bosnia and Herzegovina	23 (21.7)
Northeastern Bosnia and Herzegovina	29 (27.36)
Eastern Bosnia and Herzegovina	4 (3.77)
Herzegovina	14 (13.21)
Awareness of anthelmintic resistance	
Yes	98 (92.45)
No	8 (7.55)

**Table 3:** Results of multivariable ordinal logistic regression analyses for factors associated with therapy practices of farmers (n = 188).

Variable	OR	Std. Err.	z	*p* > |z|	95% CI
combi	49.3	86.63	1.01	0.310	[0.14–308.27]
alben	0.089	0.14	−1.21	0.227	[0.0047–1.73]
albgan	1.59	1.68	0.44	0.661	[0.20–12.68]
nuth	0.69	0.29	−0.88	0.291	[0.31–1.56]
freq	0.73	0.61	−0.38	0.705	[0.14–3.74]

**Table 4:** Results of multivariable ordinal logistic regression analyses for factors associated with management and environmental factors of farmers (n = 188).

Variable	OR	Std. Err.	z	*p* > |z|	95% CI
which	0.29	0.32	−1.11	0.267	[0.03–2.55]
altitude = 2	0.40	0.41	−0.89	0.376	[0.05–3.06]
altitude = 3	0.10	0.13	−1.83	0.067	[0.01–1.17]
qarant	0.52	0.53	−0.64	0.521	[0.07–3.80]
newdang	0.35	0.34	−1.08	0.279	[0.05–2.35]

**Table 5:** Results of Firth’s logistic regression (main variables).

Variable	OR	Std. Err.	Z	*p* > |z|	95% CI
combi	102.2	177.55	2.67	0.008	[3.42–405.01]
alben	0.092	0.14	−1.57	0.117	[0.005–1.82]
altitude = 2	0.51	0.54	−0.64	0.520	[0.06–4.02]
altitude = 3	0.10	0.13	−1.80	0.072	[0.01–1.17]

**Table 6:** Firth’s logistic regression results with quarantine variable.

Variable	OR	Std. Err.	z	*p* > |z|	95% CI
combi	98.83	172.12	2.64	0.008	[3.25–300.16]
alben	0.10	0.15	−1.53	0.126	[0.005–1.91]
altitude = 2	0.51	0.55	−0.63	0.530	[0.06–4.14]
altitude = 3	0.089	0.12	−1.86	0.063	[0.007–1.14]
qarant	0.41	0.42	−0.87	0.383	[0.06–3.01]

**Table 7:** Firth’s logistic regression results for knowledge, attitude and practice variables.

Variable	OR (95% CI)	*p*-Value
Ahrum	45.64 (1.82–1142.78)	0.020
Ahreg	0.30 (0.10–0.87)	0.027
Experience (exp)	1.53 (0.81–2.90)	0.186

Note: Firth’s logistic regression was used due to potential separation and the small sample size.

## Data Availability

All data generated in this paper are included in the manuscript.

## Abbreviations

The following abbreviations are used in this manuscript:

GIN	Gastrointestinal Nematode
AH	Anthelmintic Resistance
BZ	Benzimidazole
SNP	Single-Nucleotide Polymorphism
MLE	Maximum Likelihood Estimation
VIF	Variance Inflation Factor
TST	Targeted Selective Treatment
TT	Treating All Animals

## Appendix A

**Table A1:** Veterinarian anthelmintic control survey.

Gender	(a) Male (b) Female (c) Prefer not to specify
Age (years)	(a) 18–35 (b) 36–50 (c) 51–65 (d) Over 65
Years of experience	(a) 0–4 (b) 5–15 (c) 16–20 (d) 21–30 (e) Over 30
Canton/county in FB&H where the veterinary organization you are employed in is located	(a) Western Bosnia (b) Southwestern Bosnia (c) Central Bosnia (d) Northeastern Bosnia (e) Eastern Bosnia (f) Herzegovina
Private/public veterinary organization	(a) Private (b) Public
Are you familiar with the term “anthelmintic resistance”	(a) Yes (b) No
Does your daily veterinary practice involve administering anthelmintics to large and small ruminants	(a) Yes (b) No
The use of anthelmintics in daily practice is	(a) Therapeutic (b) Therapeutic/prophylaxis (c) Prophylaxis
Is it known to you that the administration of anthelmintics to large and small ruminants is often carried out by farmers themselves without prior consultation with veterinarians	(a) Yes (b) No
Do you use Monil 5% albendazole in treatment?	(a) Never (b) Sometimes (c) Always
Do you use Monil 1125 mg albendazole in treatment?	(a) Never (b) Sometimes (c) Always
Do you use Panacur 10% fenbendazole in treatment?	(a) Never (b) Sometimes (c) Always
Do you use Oxy-zole in treatment?	(a) Never (b) Sometimes (c) Always
Do you use Abantel abamectin in treatment?	(a) Never (b) Sometimes (c) Always
Do you use Dehelman 10% levamisole in treatment?	(a) Never (b) Sometimes (c) Always
Do you use Polivermin levamisole in treatment?	(a) Never (b) Sometimes (c) Always
Do you use Panvermin 300 mg albendazole in treatment?	(a) Never (b) Sometimes (c) Always
Do you use Albendazole 10% ganadexil in treatment?	(a) Never (b) Sometimes (c) Always
Do you monitor the registration of new anthelmintics and adjust practices accordingly?	(a) Yes (b) No
How often do you use panacur 10%, monil, panvermin, albendazol 10%, ganadexil	(a) Never (b) Sometimes (c) Always
Do you use the same product every year against parasites	(a) Yes (b) No
How many times a year do you treat large and small ruminants against parasites	(a) I do not administer treatment every year (b) 1–2 (c) 2–3 (d) 3–4 (e) 4–5 (f) more than 5
Treatment with anthelmintics for large and small ruminants is carried out during the period	(a) January–March (b) April–June (c) July–September (d) October–December
Do you approximate the dose of anthelmintics without previously weighing a specific number of animals	(a) No (b) According to Instructions (c) Yes
Do you perform any parasitological tests before administering anthelmintics to large and small ruminants	(a) Yes (b) No
Do you perform any parasitological tests after administering anthelmintics to large and small ruminants	(a) Yes (b) No
Have you ever suspected reduced effectiveness (loss of efficacy) of anthelmintics you use in large practice	(a) Yes (b) No
Have you ever carried out any assessment of the efficacy of anthelmintics in large and small ruminants	(a) Yes (b) No
Do farmers request and follow your advice regarding the treatment of animals with anthelmintics	(a) Yes (b) No
Factors you consider when choosing anthelmintics	(a) Degree of infection (b) Medication price (c) Route of administration of medication (d) Withdrawal period (e) Type of parasite (f) Experience (g) Duration of medication effect (h) Number of animals being treated
Which infections do you most commonly administer anthelmintics for	(a) GIN infections (b) Liver fluke infection (c) Ascarid infections (d) Tapeworms (e) Lung nematodes (f) Babesiosis (g) Theileriosis (h) Cryptosporidiosis (i) Coccidiosis (j) External parasites

**Table A2:** Farmer anthelmintic control survey.

Age (years)	(a) 18–35 (b) 36–50 (c) 51–65 (d) Over 65
Farm location	(a) Western Bosnia (b) Southwestern Bosnia (c) Central Bosnia (d) Northeastern Bosnia (e) Eastern Bosnia (f) Herzegovina
Is your farm located in a lowland, hill, or mountain area?	(a) Lowland (b) Hill (c) Mountain
Which animal species do you raise?	(a) Sheep (b) Goat (c) Cattle (d) Sheep and Goat (e) Sheep and Cattle
What is the number of animals on your farm?	(a) 1–10 (b) 10–20 (c) 20–30 (d) 30–40 (e) >40
Which of the offered grazing methods do you use?	(a) Permanent grazing (b) Seasonal grazing (c) Enclosed facility with or without an outdoor area
During the season, do you move animals to different pastures in search of grazing?	(a) Yes (b) No
Do you practice quarantine for newly arrived animals?	(a) Yes (b) No
Do newly arrived animals pose a risk for parasitic infections?	(a) Yes (b) No
Do you graze cattle and sheep together?	(a) Yes (b) No
Do you plan and administer antiparasitic treatment on your own, without prior consultation with a veterinarian?	(a) Yes (b) No
How many times per year do you administer antiparasitic drugs to animals?	(a) I do not perform treatment every year (b) 1 to 2 times per year (c) 2 to 3 times per year (d) 3 to 4 times per year (e) 4 to 5 times per year (f) More than 5 times per year
In which period do you administer antiparasitic drugs?	(a) January–March, (b) April–June, (c) July–September, (d) October–December
What are your observations on the effectiveness of antiparasitic drugs?	(a) I haven’t noticed, (b) I have noticed inefficiency, (c) I don’t know
Have you ever assessed the efficacy of antiparasitic drugs in any way (on your own or with a veterinarian)?	(a) Yes (b) No
Before treatment of large and small ruminants with anthelmintics, do you perform any parasitological examinations (fecal sampling to detect presence of parasites)?	(a) Yes (b) No (c) I don’t know
After treatment of large and small ruminants with anthelmintics, do you perform any parasitological examinations (fecal sampling to detect presence of parasites)?	(a) Yes (b) No (c) I don’t know
Do you use Monil 5% albendazole in treatment?	(a) Never (b) Sometimes (c) Always
Do you use Monil 1125 mg albendazole in treatment?	(a) Never (b) Sometimes (c) Always
Do you use Panacur 10% fenbendazole in treatment?	(a) Never (b) Sometimes (c) Always
Do you use Oxy-zole in treatment?	(a) Never (b) Sometimes (c) Always
Do you use Abantel abamectin in treatment?	(a) Never (b) Sometimes (c) Always
Do you use Dehelman 10% levamisole in treatment?	(a) Never (b) Sometimes (c) Always
Do you use Polivermin levamisole in treatment?	(a) Never (b) Sometimes (c) Always
Do you use Panvermin 300 mg albendazole in treatment?	(a) Never (b) Sometimes (c) Always
Do you use Albendazole 10% ganadexil in treatment?	(a) Never (b) Sometimes (c) Always
Other benzimidazole-based drugs not listed above	(a) Never (b) Sometimes (c) Always
Which of the listed registered veterinary drugs do you use to treat large and small ruminants for parasites	(a) Never (b) Almost never (c) Almost always (d) Always
Do you combine two or more antiparasitic drugs?	(a) Yes (b) No (c) I don’t know
How often do you use panacur 10%, monil, panvermin, albendazole 10%, ganadexil?	(a) Never (b) Almost never (c) Almost always (d) Always
Do you use the same parasitic treatment every year?	(a) Yes (b) No (c) I don’t know
Do you follow veterinarian’s advice when using antiparasitic drugs?	(a) Yes (b) No
Do you estimate the required drug dose approximately (roughly)?	(a) Yes (b) No (c) I don’t know

**Table A3:** List of survey questions and corresponding variables for veterinarians.

Questions	Variables
Veterinarians	vet
Sex	sex
Age	age
Years of experience	exp
Canton in the Federation of Bosnia and Herzegovina where the veterinary organization you work for is located	region
Private/public veterinary organization?	pripub
Are you familiar with the term anthelmintic resistance?	knowahr
Does your daily veterinary practice involve administering anthelmintics to large and small ruminants?	ahrum
In your daily practice, the application of anthelmintics is?	ahapp
Are you aware that the administration of anthelmintics to large and small ruminants is often carried out by farmers themselves without prior consultation with a veterinarian?	farmahapp
Do you use Monil 1125 mg albendazole in treatment?	alben2
Do you use Panacur 10% fenbendazole in treatment?	fenben
Do you use Oxyzole in treatment?	oxyzl
Do you use Abantel abamectin in treatment?	abam
Do you use Dehelman 10% levamisole in treatment?	dehlev
Do you use Polivermin L levamisole in treatment?	polilev
Do you use Panvermin 300 mg albendazole in treatment?	alben3
Do you use Albendazole 10% Ganadexil in treatment?	albgan
Other benzimidazole-based drugs not listed?	othbenz
Do you follow the registration of new anthelmintics and adjust your practice accordingly?	ahreg
How often do you use Panacur 10%, Monil, Panvermin, Albendazole 10%, Ganadexil?	freq
Do you use the same antiparasitic product every year?	same
How many times per year do you treat large and small ruminants against parasites?	freqyear
You administer anthelmintics to large and small ruminants during the period?	perth
Do you estimate the dose of anthelmintics approximately, without prior weighing of a certain number of animals?	dose
Before administering anthelmintics to large and small ruminants, do you perform any parasitological examinations?	scbef
After administering anthelmintics to large and small ruminants, do you perform any parasitological examinations?	scaft
Have you ever suspected reduced efficacy (loss of effectiveness) of the anthelmintics you use in large-scale practice?	effectloss
Have you previously assessed the efficacy of anthelmintics in large and small ruminants in any way?	effectest
Do farmers seek and follow your advice regarding antiparasitic treatment of animals?	advice
Factors you consider when choosing an anthelmintic?	choice
For which infections do you most often administer anthelmintics?	inftype

**Table A4:** List of survey questions and corresponding variables for farmers.

Questions	Variables
Farmers	farm
Age of the farmer	age
Farm location	loci
Is your farm located in a lowland, on a hill, or in the mountains?	altitude
Which type(s) of animals do you raise?	spec
What is the total number of animals on your farm?	nums
Which grazing system(s) do you apply?	graz
During the grazing season, do you move animals to different pastures in search of forage?	move
Do you practice quarantine for newly introduced animals?	qarant
Do you consider newly introduced animals a potential risk for parasitic infections?	newdang
Do you graze cattle and sheep together?	cohab
Do you independently plan and administer antiparasitic treatments without prior consultation with a veterinarian?	plth
How many times per year do you administer antiparasitic treatments?	nuth
In which period(s) of the year do you administer antiparasitic treatments?	perth
What are your observations regarding the effectiveness of antiparasitic drugs?	obsth
Have you ever assessed the efficacy of antiparasitic drugs (either independently or with the assistance of a veterinarian)?	estith
Before administering anthelmintics to small or large ruminants, do you perform any parasitological diagnostics (e.g., fecal examination for parasite presence)?	scbef
After administering anthelmintics to small or large ruminants, do you perform any parasitological follow-up diagnostics (e.g., fecal examination for parasite presence)?	scaft
Do you use Monil 5% albendazole in treatment?	alben
Do you use Monil 1125 mg albendazole in treatment?	alben2
Do you use Panacur 10% fenbendazole in treatment?	fenben
Do you use Oxyzole in treatment?	oxyzl
Do you use Abantel (abamectin) in treatment?	abam
Do you use Dehelman 10% levamisole in treatment?	dehlev
Do you use Polivermin L (levamisole) in treatment?	polilev
Do you use Panvermin 300 mg albendazole in treatment?	alben3
Do you use Albendazole 10% Ganadexil in treatment?	albgan
Do you use any other benzimidazole-based drugs not listed above?	othbenz
Which of the listed registered veterinary drugs do you use to treat large and small ruminants against parasites?	which
Do you combine two or more antiparasitic drugs during treatment?	combi
How frequently do you use Panacur 10%, Monil, Panvermin, Albendazole 10%, or Ganadexil?	freq
Do you use the same antiparasitic drug every year?	same
Do you follow veterinary recommendations when applying antiparasitic drugs?	advice
Do you estimate the dose of antiparasitic drug approximately (i.e., by visual assessment)?	dose
Has resistance or the presence of resistance genes been confirmed on your farm?	resist
